# ‘It’s Not About the Food’—Understanding the Lived Experience of Patients Who Developed Hospital-Acquired Malnutrition (HAM) and That of Their Carers

**DOI:** 10.3390/healthcare14121806

**Published:** 2026-06-22

**Authors:** Michelle Palmer, Angela Vivanti, Breanne Hosking, Fiona Naumann, Sally Courtice, Amanda Henderson, Hazel Harden, Shoni Philpot, Anne Smyth, Lynda Ross

**Affiliations:** 1 Nutrition and Dietetics, Logan Hospital, Queensland Health, Meadowbrook, Logan City, QLD 4131, Australia; breanne.hosking@health.qld.gov.au (B.H.); fiona.naumann@health.qld.gov.au (F.N.); 2 Research and Development Dietitian, Department Nutrition and Dietetics, Princess Alexandra Hospital, Queensland Health, Brisbane, QLD 4102, Australia; angela.vivanti@health.qld.gov.au; 3 School of Human Movement and Nutrition Studies, University of Queensland, Brisbane, QLD 4072, Australia; 4 Nutrition and Dietetics, Beaudesert Hospital, Queensland Health, Beaudesert, QLD 4285, Australia; 5 Nutrition and Dietetics, QEII Hospital, Queensland Health, Coopers Plains, Brisbane, QLD 4108, Australia; sally.courtice@health.qld.gov.au; 6 Nursing Practice Development Unit, Princess Alexandra Hospital, Queensland Health, Ipswich Road, Woolloongabba, Brisbane, QLD 4102, Australia; amanda.henderson@health.qld.gov.au; 7 School of Nursing, Midwifery and Social Sciences, Central Queensland University, Rockhampton, QLD 4701, Australia; 8 Anne Smyth Organisational Consulting, Camberwell, Melbourne, VIC 3124, Australia; hazeleharden@gmail.com (H.H.); shoni.philpot@hotmail.com (S.P.); anne@ldc.net.au (A.S.); 9 School of Exercise and Nutrition Sciences, Queensland University of Technology, Kelvin Grove, Brisbane, QLD 4059, Australia; l20.ross@qut.edu.au

**Keywords:** Hospital-Acquired Malnutrition, experiences, perceptions, carers, patients

## Abstract

*Background/Objectives:* Given the limited evidence internationally, this qualitative study employed discovery interviews to explore the lived experience of patients who developed Hospital-Acquired Malnutrition (HAM) and that of their carers. *Methods:* Seven (two patients [(*n* = 1 female] and five carers [*n* = 3 female]) completed discovery interviews with an experienced independent interviewer. Carers were either spouses or parents. Responses were thematically analyzed using a constant comparative approach. *Results:* A key theme was ‘It’s not about the food, it’s the hospital system’ with the needs of the system dominating, including when patients were feeling at their worst. Subthemes were ‘integration of care’ and ‘patient acuity’, including symptoms that impacted food intake. Another theme was ‘Who is looking out for the patient?’, exploring ‘reliance on carer advocacy’, and ‘variation in staff involvement’. One carer said, “… the girl that delivered the meal tray was the only one in our hospital stay who actually said to [the patient], ‘I’m so glad you’re sitting up. I was worried about you because you hadn’t eaten for so long?” A persistent but comparatively less strong theme was ‘When it is about the food’ which explored ‘the quality of the food’ and ‘receiving information on eating and drinking’. *Conclusions:* The three key themes identified from carers and patients were hospital system impacts, care co-ordination and, less strongly, experiences with food quality and information. The key opportunities to prevent, or better support the nutritional care of patients with, HAM may be through improving systems and care co-ordination.

## 1. Introduction

Hospital-Acquired Malnutrition (HAM) is officially recognized as a hospital-acquired complication in Australia [1]. Currently the definition of HAM varies internationally. Definitions include patients who were first diagnosed with malnutrition > 14 days after hospital admission [2,3], and any decline in nutritional status during hospital stay [4]. Given HAM is a recognized hospital-acquired complication, understanding the perspective of those with lived experience of HAM may assist in improving nutrition-related hospital care, which is important to consumers.

Internationally, the authors did not locate any studies that sought the experiences, opinions, or insights of either patients who developed HAM or of their carers. Similar nutrition-related qualitative inpatient studies (hereafter referred to as similar studies) generally did not report patients’ nutritional status [5,6,7,8,9,10,11,12,13,14,15], and the remainder that did generally reported the risk of malnutrition [16,17,18], with only one diagnosing malnutrition [19]. Further, few similar studies specifically interviewed long-stay patients (i.e., >14 days) [19] or carers [6,7,8,13,18]. Common themes emerging from these similar studies included the impact of nutrition impact symptoms such as poor appetite and taste and smell changes on eating [6,8,9,10,11,12,14,15,16,17,18,19,20]. Foodservice themes were also commonly reported, including the importance of menu variety, adequate portions, and the acceptability and appearance of the foods delivered [8,10,11,12,13,14,18,21]. Protected mealtimes were also regularly identified, including the impact of mealtime interruptions, and patients who required mealtime assistance either not receiving it or having to wait as staff were busy [5,9,10,11,13,14,15,18,20,21]. Patients also routinely reported that social support during mealtimes was important [9,11,12,15,18,19]. Less common themes included hospital systems [5,15] and a fragmented approach to care [9] impacting on the ability to eat. While a variety of themes were reported in the relevant literature, no studies were located that addressed the insights of either longer-stay patients with HAM or of their carers.

To enhance understanding of nutritional care delivery of longer-stay patients, this study used a qualitative design incorporating a narrative thematic analysis and comparative approach and employed discovery interviews to explore the lived experience of patients who developed HAM and that of their carers.

## 2. Methods

### 2.1. Study Design, Ethics, Location

Carers and patients who had been discharged from one of five facilities (*n* = 4 urban, *n* = 1 regional) located in Southeast Queensland, Australia, were recruited for discovery interviews. The Metro South Human Research Ethics Committee approved this study (HREC/2022/QMS/81577) prior to commencement. Informed consent was obtained from participants. The HAM definition used in this study was malnutrition first diagnosed >14 days after hospital admission, inclusive of consecutive lengths of stay from transferring hospital(s) [2,3]. A consumer reviewed the proposed project prior to study commencement.

### 2.2. Eligible Participants

The eligible participants were adults (≥18 years), either patients admitted to a Metro South hospital between August 2023 and February 2025 on any ward (including intensive care, acute, sub-acute or mental health) who were diagnosed with HAM by a dietitian (hereafter referred to as ‘a patient with HAM’) or their carers, who consented to participate and be recorded during the interview. Dietitians across these facilities diagnosed malnutrition using the Subjective Global Assessment [22]. A ‘carer’ in this instance refers to any significant other of the patient who was engaged in the patient’s care while in hospital and spent many days at their bedside.

Patients and carers were excluded if: the patient was palliative (or on the trajectory of the palliative pathway), the patient had an intellectual, cognitive or mental impairment, the patient or carer was unable to provide an accurate history (e.g., cognitive impairment, or the patient was catatonic during their mental health stay), or were unable to communicate in English either in writing or verbally.

### 2.3. Data Collection

Purposive sampling [23] was used to target the population of interest. Eligible participants were located through both the hospital systems for documentation of HAM and the clinicians alerting the research team through maintained departmental awareness of the study. Potential participants were invited to participate during their hospital admission. If they verbally consented, their contact details were provided to an independent, external interviewer not involved in patient care with expertise in conducting discovery interviews. The interviewer then contacted the potential participant to discuss the research and arrange an interview. To limit bias in data collection and interpretation, the research team was then blinded to the identity of participants, except for the two external members conducting the interviews. The interviewers were two adult females not involved in the care of the patients who were experienced in conducting discovery interviews.

The obtaining of informed consent and the interviews were conducted at a time and location convenient to participants and either conducted during the admission (*n* = 1) or post-discharge. Telehealth was utilized to conduct interviews if required and convenient for the participant. Participants were advised when the interview recording was turned on and off. If there were any interruptions to the interview, e.g., another family member entering the room, the recording was turned off during this time. Interviews were all conducted separately (for example, in the case of a patient and carer who lived together) to ensure the confidentiality of any comments made by either party and to ensure that their view remained independent of the other person. Interview recordings were transcribed verbatim. The primary outcome was a collection of carer and patient stories from which themes were derived about the hospital care provided that may have influenced HAM.

**Figure 1:**
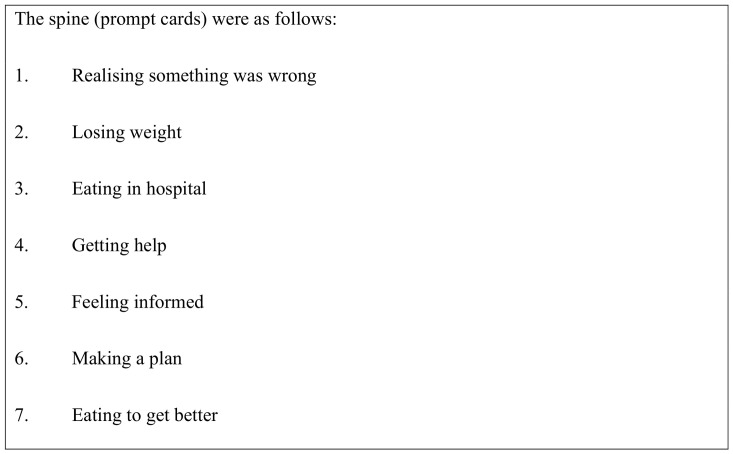
The interview spine used during all discovery interviews with eligible participants.

Discovery interviews (DIs) are a method consistent with the qualitative approach taken here, which were developed in the NHS and are widely used to explore patient journeys and subjective experiences. They take a discovery and exploratory approach and do not direct or pre-empt interviewee responses. Rather, their comparatively open structure enables participants to focus on and explore aspects of their experience they think are relevant and important [24,25]. The discovery interview method assists participants with sharing an open “story” of their health care experience using only core prompts (called an interview “spine”) facilitated by cards with key words or phrases developed by researchers and clinicians and reviewed by a consumer representative [24]. The spine was introduced to the participant with an explanation of the project to provide context. The spine consisted of seven prompt cards (see Figure 1) intended to assist participants to tell their story. All cards were displayed in front of participants (if in person) or on screen if via telehealth and placed in no particular order. Participants chose the prompt card they wanted to discuss first. The interviewer guided participants to cover all the prompt cards if they wished to. The interviewer interrupted participants only to ask them to expand on an experience they raised. Prompts or probing questions were only used to encourage participants to continue telling their story. The discovery interview technique identifies “…a small number of men and women, who are typical rather than an exact representative sample” [24]. It provides a narrative to be discussed and explored with clinicians in the context of the patient’s experience with food and mealtimes. Narrative material sufficient for this purpose can therefore be developed from a small number of patient stories.

### 2.4. Analysis

Thematic analysis was completed by an experienced qualitative researcher using Microsoft Word and confirmed by the research team. Narrative thematic analysis was applied to identify and interpret key patterns within the data, while the comparative method enabled examination of similarities and differences across interviews. Themes were developed by discussing the dimensions and properties for each participant. This occurred within and across interviews which were analyzed individually and then as a whole data set. Codes were identified and grouped into themes which were moved up and down levels of abstraction throughout this process by the analyst [26]. Triangulation occurred through the involvement of a second investigator verifying interpretation [27]. The research team then discussed and refined the final thematic categories over several meetings.

The transcripts of interviews were analyzed in two stages. In stage one, the interview transcripts were edited (without changing the words of the patient) to generate a narrative. In the second stage, participants’ narratives were analyzed to build up robust descriptions, as patients and carers saw it, of the experiences of eating and mealtimes during hospital admission. Then, through an iterative cross-narrative approach, sense was made of the descriptions by developing higher-level themes [28]. Each theme was evidenced by verbatim examples from the narratives to ensure the voice of informants was paramount and provided a clear and robust chain of evidence from the words of informants to the interpretations of the researcher.

The themes identified described what matters to carers and patients regarding eating and mealtimes and provide insights into the reason(s) for decreased food intake, eating experience, and organizational and environmental barriers to eating in hospital. Both positive and negative experiences were explored.

Each interview was analysed to identify key codes, with subsequent interviews examined for new dimensions, nuances, or insights related to those codes. Drawing on Hennink’s framework [29], saturation was reached when no further meaningful elaboration of existing codes emerged, emphasising depth, interpretive richness and understanding rather than large sample size.

The strength of the theme was based on both its frequency of occurrence and the extent to which patients and carers conveyed its significance and impact on their experiences. Particular attention was given to the content of participants’ accounts, the language they used, and the cadence of their narratives, which served as important guides in the development and refinement of themes. Patient and carer quotes were selected to demonstrate the key themes [30]. Coding of quotes occurred sequentially from the first patient coded as Pt1, with their carer coded as Carer1, and then as Carer1a and Carer1b if two carers relating to the same patient were interviewed.

## 3. Results

Twenty-nine carers or patients were initially approached. Twenty verbally consented, and seven (two patients [(*n* = 1 female] and five carers [*n* = 3 female] 35%, *n* = 7/20) were subsequently interviewed. All participants interviewed were from the same hospital. The caring relationship included spouses (*n* = 2) and parents (*n* = 3). Both patients interviewed also had a related carer who was interviewed. Three carers who were interviewed represented an additional two patient experiences; however, those related patients declined an interview. Two carers reported a health background. The patients and carers interviewed represented a range of experiences from general hospital to surgical and rehabilitation wards. Interview duration ranged from 45–65 min.

The thematic analysis of the interviews highlighted three major themes and several sub-themes (Table 1). The first two themes were extremely strong and were discussed across all interviews. The third theme, while not unimportant, was not as consistently emphasized by patients and carers. Overall, the themes that emerged were consistent within each interview and across the data set and were generated until saturation of themes was achieved and no additional meaningful understandings emerged. Considerable commonality existed between related patient and carer interviews which justified the combining of their accounts. They were only distinguished by the subtheme of high reliance on carer advocacy, and even then this was a common view of both patients and carers.

### 3.1. Theme 1: It’s Not About the Food, It’s the Hospital System

This was a major theme and whilst other themes were important, the impact of the hospital system was far more prominent. The needs of the system dominated and were reported by informants as resulting in absent or delayed clinical responses including nutritional care, and lack of provision of information, care and support for patients and their carers when they needed it.

*“I personally felt like at times you were just inconveniencing the system.”*

(Pt1, male)

*“It frustrates me that the system’s so broken and we live in this amazingly wealthy country … where huge money is spent on health care and we’re getting it so wrong.”*

(Carer1, female)

#### 3.1.1. Subtheme 1: Integration of Care—Lack of Co-Ordination of Care and Engagement with Patient/Carer

If, and when, advice did arrive it was often perceived as unhelpful, with the co-ordination of care between different clinicians frequently described as poor and characterized by failure to notice significant weight loss and hunger, disorganization and fragmentation, and limited engagement and communication. This left patients and their carers stranded. Carers had to work it all out for themselves and actively advocate for attention and action.

*“Poor handovers, poor communication. No one in charge. No one.”*

(Carer1, female)

*“There didn’t seem to be anyone in charge of pain control. They indicated that he was quiet so that’s why no one was sort of paying attention.”*

(Carer1, female)

*“Umm, you know, marking off all the obs and getting all that done and it just felt like at times it wasn’t really looking at the patient. And I know one day the computer system crashed…, you could see people were in a panic! They were lost because the computers weren’t working…It was like the sky falling…”*

(Pt1, male)

*“…it kinda felt like, yeah, you’re just sort of taking up their time…”*

(Pt1, male)

*“…their care was focused [on brain injuries]. …eating and nutrition wasn’t just so much on their plan…”*

(Carer2, male)

*“…getting help was hard because we didn’t feel like we were listened to at all”*

(Carer1, female)

These challenges resulted in a range of consequences although they were not the same in all cases. The consequences included delayed attention to nutrition issues, sometimes poor and fragmented responses, little follow up, inconsistent practices between wards, a sense of chaos at times, apparent clinical risk and little help with nutrition planning upon discharge—an important component of sustaining recovery and supporting patients and the family.

*“The menu could have been great, but of no benefit if there was not someone there, in some cases to make sure the person has a) ordered appropriately in the first instance and b) finished their meal.”*

(Carer2, male)

*“…the day of discharge, …the dietitian…said she only heard about me the day before…she was shocked…”*

(Pt1, male)

*“I left hospital and there was no plan for me. I just felt like they had nothing.”*

(Pt1, male)

However, occasionally it was acknowledged that the system was working well.

*“Yeah, so you all got support. Like help from everyone.”*

(Carer4b, male)

#### 3.1.2. Subtheme 2—Patient Acuity—The Acuity and Nature of the Patient’s Clinical Condition

The carers’ and patients’ stories clearly highlighted that they were feeling unwell in hospital, including experiencing life-threatening conditions and many common nutrition impact symptoms such as pain, tiredness, and fluid retention. These challenges all impacted whether nutrition was prioritized by the patient and care team, the patient’s appetite and their ability to eat, and how much care might be required to assist them to get adequate nutrition. Delays to treating these conditions can also further delay receiving adequate nutrition and result in weight loss. Participants observed that often these clinical signs appeared not to be noticed and acted upon.

*“…He had to learn again how to eat and swallow. So that was a bit of a worry, about the amount of weight and how quickly he lost it was a concern…”*

(Carer3, female)

*“he had poor pain management… And that pain and nausea…impacted on his ability to eat and no one was really listening to that, and so the pain got worse and he didn’t eat.” “…So probably three days, he was managing to eat only a couple of things. His pain got out of control on day three or four, when the hospital’s computer systems went down, and I think they lost track of his pain control.” “he was starving…” “Day nine is when we finally got it sorted.”*

(Carer1, female)

However, participants acknowledged and were reassured when there was proactive, coordinated care.

*“She goes up and sees them and they do their blood tests, and they check her…they need to see a visual and make sure that she’s looking well and everything is well. To reflect what the bloods are saying, they need to see that.”*

(Carer4a, female)

*“But the dietitian up there was really good. She said, well, I have another menu too, and high protein or mineral I think.”*

(Carer4b, male)

### 3.2. Theme 2: Who Is Looking out for the Patient?

While all staff can monitor nutritional intake, the carers and foodservice staff (for example, staff delivering meal trays) were observed to be looking out for the patients, including their eating and drinking. This was instead of the expected ward clinicians noticing the changing nutrition situation. Dietitians’ input was noted by interviewees as absent, limited or delayed.

#### 3.2.1. Subtheme 1: High Reliance on Carer Advocacy—The Pivotal Role of Carers and Their Advocacy

Carers played a large, essential role in every aspect of the nutritional care of their family member, including identifying significant weight loss, lack of patient engagement with eating, and issues contributing to this. Carers often addressed what they noticed, alerted clinicians and followed through on responses. This usually required persistence and sometimes sheer hard work.

*“If [the carer] hadn’t been there I’d be not good at all like because I would have been in a lot of pain, a lot longer. And no one spoke about my weight at all and yeah, she was pretty much my voice the whole time through it.”*

(Pt1, male)

*“I mean for me, it’s okay because my husband is going in telling me eat this sort of thing. But I feel like if I didn’t have that, you just didn’t have a clue. You feel a little bit powerless.”*

(Pt2, female)

*“I see a lot of older people and I’ve said to them, probably what I learned from that experience was, have an advocate, push harder. Don’t be silent…”*

(Carer1, female)

Carers acknowledged when care was done well and could see the demands the clinicians were under. However, this was not common except in two examples of exemplary nutritional care where the service design featured multidisciplinary teams and well-integrated care throughout the patient journey.

*“I thought everyone [on the ward] made that so easy for us. …we talked about a lot (making a plan) and actually because [the carer] and someone else…and [the patient] and whoever else was involved, I was involved, worked with everyone. …there was no sort of conversation about [the patient] that we didn’t involve [the patient] with.”*

(Carer3, female)

#### 3.2.2. Subtheme 2: Healthcare Staff Involvement and Influence Varied

Instead of the expected clinicians noticing the nutrition situation, it was left to foodservice staff and carers. One carer noted that clinical staff appeared to have their heads in electronic devices.

*“…no one noticed him… on about day 10 or 11, the girl that delivered the meal tray was the only one in our hospital stay who actually said to [the patient], ‘I’m so glad you’re sitting up. I was worried about you because you hadn’t eaten for so long?”*

(Carer1, female)

*“If the person didn’t complete the menu appropriately because they weren’t functional… It would be really helpful if you could at least help to make sure she eats at least a reasonable amount of food, particularly high-protein foods.”*

(Carer2, male)

*“I just have vivid memories of people just being so glued to bloody screens the whole time.”*

(Pt 1, male)

Perceptions of the dietitians’ services ranged from being almost missing in action, having limited input and options to offer, not always following through (for example after weight checks), being supportive but not seeming to add much value, and in the two exceptional cases, being a core part of a team and of real value.

*“I believe that I saw the dietitian in the discharge lounge on Day 14 and I believe she was only notified of my existence on Day 13, right? …she was pretty shocked at the condition I was in…”*

(Pt1, male)

There was a sense, sometimes explicitly observed, that patients and carers were not receiving adequate information to address specific needs at various stages of recovery and for carers to support them.

*“…we don’t get told about how important nutrition is.”*

(Pt2, female)

### 3.3. Theme 3: When It Is About the Food

The quality and quantity of food and information and guidance about food did receive comment but was not as strong a theme as the other themes.

#### 3.3.1. Subtheme 1—Food Considerations—The Quality and Quantity of Food

While the quantity was generally acceptable, the quality was perceived as just OK, and the meals were repetitive especially if the hospital stay was long. In several cases, carers played a significant role in sourcing and supplementing diets with high-energy, high-protein food to address weight loss and responding to their family members who reported feeling very hungry.

*“Hospital food, it can be a bit repetitive, and I have to say there was one chicken curry I had, it was awesome…there was some variety, but sometimes you just look at it and go it’s not very appealing. [The carer] did buy quite a bit of food and left it in the fridge on the ward…”*

(Pt1, male)

*“…it wasn’t really particularly appetizing, coupled with the fact, her smell was certainly very, very compromised, and to be fair, I had a look at some of the food with all due respect there and the easy chew food doesn’t look wonderful so she didn’t eat much. That would have likely been one of the contributors to her weight loss.”*

(Carer2, male)

*“…And no one checks like when they give you the meal, no one goes, oh, you’ve just got a smear of sweet potato, are you sure you don’t want something else?”*

(Pt2, female)

Participants acknowledged that in large hospital systems providing quality and appealing meals for larger numbers of unwell people with varying requirements was a very hard task.

*“But they do well. I mean there’s a lot of people here and you can imagine how many different requirements there are in every room and every ward…”*

(Pt2, female)

#### 3.3.2. Subtheme 2: Receiving Information and Guidance on Eating and Drinking

The patients and carers involved felt lost and uncertain regarding eating and drinking and in most cases received limited guidance. One patient highlighted the importance of education and felt there were opportunities to run groups in hospital to address this need. Concepts expressed included understanding the nutritional needs of someone who had their particular condition, what was needed to recover properly, how food interacted with ongoing medication and the role of exercise. There was a sense, sometimes explicitly observed, that patients and carers were not receiving adequate information to address specific needs at various stages of recovery and for carers to support them. The areas of greater concern revolved around the kind of nutrition that that was appropriate for someone who had been very ill and lost a great deal of conditioning and weight.

*“There’s no plan. I don’t think that existed. Even on discharge, there was no plan… On the discharge plan, it was even written down that he didn’t need to visit the GP for follow up… it would have been nice to have a laid-out plan…”*

(Carer1, female)

*“So, getting the plan, getting the dietitian there and getting it onto a normal diet was a good plan…”*

(Carer2, male)

## 4. Discussion

For the first time, the lived experience, perspective and insights of patients after developing Hospital-Acquired Malnutrition and that of their carers have been reported. These views aid our understanding of factors impacting nutrition-related care of longer-stay patients who nutritionally deteriorate and what is important to carers and patients. Hospital systems, care co-ordination, patient acuity, the role of carer advocacy, and healthcare staff involvement played a more dominant role in carer and patient experiences, with food quality and guidance representing a minor theme. Preventing and managing nutrition-related deterioration requires more than examining food provision but rather requires addressing the systems and clinical care provided by a range of healthcare professions.

The carers’ and patients’ stories clearly highlighted that the needs of the hospital system dominated, and that the coordination of care (or lack of) impacted the quality of patient’s clinical and nutrition-related care. Most similar studies focused specifically on the impacts that the foodservice system can have, including meal timing, delivery processes, and choice [8,9,10,11,12,13,14,18,19,20,21], and co-ordination of care at meal times in terms of limiting interruptions, and assisting with meals [5,9,10,11,13,14,18,20,21]. However, a theme from two Australian studies of older (≥65 years) inpatients was “…the inflexibility of the food service and wider systems within the wards impacted on their ability to eat” [5,15]. Additionally, Scottish intensive care patients reported “…inter-related system breakdowns during the nutritional recovery process”, including system-centered failures, an inflexible hospital routine and communication failures leading to a fragmented approach to care [9]. Ross et al. proposed redefining multidisciplinary roles to support coordinated nutrition care [31]. Our findings suggest that preventing and managing malnutrition in hospital involves addressing hospital systems, which is far broader than foodservice systems and care at mealtimes only, along with care coordination.

The patient’s high level of acuity, clinical condition and nutrition impact symptoms negatively impacted their eating and drinking in hospital. Nutrition impact symptoms are known barriers to intake in patients with Hospital-Acquired Malnutrition [4]. Similar studies commonly reported that nutrition impact symptoms (e.g., nausea and vomiting, fatigue, difficulty swallowing, pain) impacted hospital intake [5,6,8,9,11,12,14,15,16,17,18,19,20]. Identifying and managing nutrition impact symptoms, particularly at mealtimes, and alleviating symptoms associated with high patient acuity and the patient’s clinical condition could facilitate eating and drinking in hospital.

The second major theme was “Who is looking out for the patient?”, with a subtheme of the pivotal role of carers and their advocacy. Carers’ advocacy partly reflected their interest in patient wellbeing and willingness to participate, but more notably, carers consistently played an essential, highly active and influential role in managing and driving the patient’s nutritional care across all interviews. Few similar studies interviewed carers. The carer role was acknowledged, particularly in terms of providing assistance with and monitoring mealtimes and providing social support, care when nursing staff were busy, and food from home [8,12,13,15,18]. A 2016 literature review of carer engagement in the hospital care of older people showed similar focal points for carers, including patient caregiving, information sharing, shared decision-making, carer feedback and patient care transitions [32]. The inclusion of carer interviews for small, unwell cohorts may assist with understanding the care journey for these patients.

The second subtheme of “Who is looking out for the patient?” was that healthcare staff involvement and influence varied. A range of staff, including dietitians, nursing, allied health and foodservice staff, were mentioned in the interviews, supporting the premise that all staff members can assist in the prevention and management of malnutrition [31]. Care rationing may occur on busy wards and contribute to variation in patient care when urgent clinical tasks are prioritized over patients’ eating and drinking needs [33]. Similar studies observed insufficient nutritional care responsibility and communication between professions, and clinical staff being busy or absent at mealtimes; however, patients valued assistance when provided, and care varied across the hospital admission [8,10,11,12,13,14,18]. Redesigning care systems to support symptom management and oral intake and including patients and carers in the care process is both a challenge and priority for acute healthcare settings.

The third theme “When it is about the food…” was not as strong as the other themes and considered the quality, repetition and quantity of food provided, along with receiving information and guidance on eating and drinking. Similar studies highlighted consistent themes around food quality, quantity, sufficient choice and food brought in from home [8,10,11,12,13,14,15,18,21]. These studies also noted that either increased patient awareness of their nutritional status, nutrition knowledge and skills influenced intake [11,15,19], or that participants reported not being aware of their risk of malnutrition and requested nutrition-related education and information [6,7,8,10,16,18,20]. The most effective messages for communicating both the diagnosis and impact of malnutrition upon patient health and outcomes need to be ascertained along with optimal strategies for disease prevention [34].

This study had several strengths and limitations. Study strengths include the use of experienced and independent interviewers, and the use of general interview prompts rather than targeted prompts that pre-empt the main themes. This serves to gather unbiased patient stories. There were also no restrictions on patient age and dietary type. Additionally, while the definition of HAM varies and has included any nutritional deterioration in hospital [4], the current findings remain relevant, as all participants experienced nutritional decline during their hospital admission. The themes observed were also consistent with current hospital standards [35], suggesting that hospital standards can facilitate nutrition-related care and are aligned with what is important to consumers. Several interviewees also demonstrated a better understanding of the health system, which provided more nuanced insights. Despite the 18-month data collection period, only seven people were interviewed who were from the same hospital. This could be due to the low prevalence of patients developing HAM [36]. While the reasons for declining interviews were not specifically sought, the high acuity that these patients experienced [13] may have led to a low interview response rate (<40%) and reliance on carers to interview. Importantly, considerable commonality existed between related patients and carers, such that carers’ reports were equivalent to patient reports. Holst et al. [17] also had a similar sample size (*n* = 12) and interviewed patients at severe nutritional risk but with a shorter hospital stay. The discovery interview method seeks deep and powerful accounts that capture the significance of the experience from the informants’ perspective. In this way, the sample reflected information power [37]. Additionally, the themes that emerged were also consistent across each interview. The hope is that this information may help inform future work in this newly developing field and help create hypothesis testing in clinical settings. Further interviews from patients and their carers would also be valuable to determine if any other themes emerge in different locations.

## 5. Conclusions

Although food quality can impact the nutrition-related care and experiences of high-risk patients, the key themes were hospital systems, care co-ordination, patient acuity and symptom control, and the role of healthcare staff and carers. Our findings showcase what is important and relevant to carers and patients during an admission where the patient develops HAM. Our findings point to opportunities to develop novel co-designed practices that prevent or better support the nutritional care of patients with HAM by changing the service design to one of coordinated, multidisciplinary systematized team care that more readily engages and empowers patients and their carers.

## Figures and Tables

**Table 1:** Main themes and subthemes of nutrition-related experiences from carers and patients who were diagnosed with Hospital-Acquired Malnutrition during a recent hospital admission.

Theme	Subtheme
It’s not about the food, it’s the hospital system.	Integration of care—Lack of coordination of care and patient/carer engagement
	Patient acuity—The acuity and nature of the patient’s clinical condition
Who is looking out for the patient?	High reliance on carer advocacy—The pivotal role of carers and their advocacy
	Healthcare staff involvement and influence varied
When it is about the food… *	Food considerations—The quality and quantity of food
	Information and guidance on eating and drinking

Key: * = This was a comparatively less strong theme, relative to themes 1 and 2.

## Data Availability

The data presented in this article are not readily available because there has no been prior ethical approval to make this data available to external parties. Requests to access the datasets should be directed to the corresponding author.
